# Human Papillomavirus Mutational Insertion: Specific Marker of Circulating Tumor DNA in Cervical Cancer Patients

**DOI:** 10.1371/journal.pone.0043393

**Published:** 2012-08-24

**Authors:** Maura Campitelli, Emmanuelle Jeannot, Martine Peter, Emmanuelle Lappartient, Stéphanie Saada, Anne de la Rochefordière, Virginie Fourchotte, Séverine Alran, Peter Petrow, Paul Cottu, Jean-Yves Pierga, Olivier Lantz, Jérôme Couturier, Xavier Sastre-Garau

**Affiliations:** 1 Department of Radiation Oncology, Institut Curie, Hospital, Paris, France; 2 Department of Biopathology, Institut Curie, Hospital, Paris, France; 3 Department of Surgical Oncology, Institut Curie, Hospital, Paris, France; 4 Department of Radiology, Institut Curie, Hospital, Paris, France; 5 Department of Medical Oncology, Institut Curie, Hospital, Paris, France; IPO, Inst Port Oncology, Portugal

## Abstract

**Introduction:**

In most cases of cervical cancers, HPV DNA is integrated into the genome of carcinoma cells. This mutational insertion constitutes a highly specific molecular marker of tumor DNA for every patient. Circulating tumor DNA (ctDNA) is an emerging marker of tumor dynamics which detection requires specific molecular motif. To determine whether the sequence of the cell-viral junction could be used in clinical practice as a specific marker of ctDNA, we analyzed a series of cervical cancer patient serums.

**Methods and Findings:**

Serum specimens of 16 patients diagnosed with HPV16/18-associated cervical cancer, and for which the viral integration locus had been previously localized, were analyzed. Sequential serum specimens, taken at different times during the course of the disease, were also available for two of these cases. ctDNA was found in 11 out of 13 patients with tumor size greater than 20 mm at diagnosis, and analysis of sequential serum specimens showed that ctDNA concentration in patients serum was related to tumor dynamics.

**Conclusions:**

We report that HPV mutational insertion constitutes a highly specific molecular marker of ctDNA in HPV-associated tumor patients. Using this original approach, ctDNA was detected in most cervical cancer patients over stage I and ctDNA concentration was found to reflect tumor burden. In addition to its potential prognostic and predictive value, HPV mutation insertion is likely to constitute a new molecular surrogate of minimal residual disease and of subclinical relapse in HPV-associated tumor. This is of major importance in the perspective of specific anti-HPV therapy.

## Introduction

Specific types of human papillomaviruses (HPV) are recognized as the major etiological factor in cervical neoplasia [1]. In pre-invasive lesions, the viral genomes are present as episomal molecules in the nucleus of infected cells. In most cases of invasive carcinoma, however, HPV DNA sequences are integrated into the cellular genome [2], [3], [4]. Viral genome integration thus represents a crucial step in tumorigenesis [5]. A single unique viral integration site has been found in more than 80% of cervical cancers [6].

The characteristic clonality, specificity and stability over time of HPV DNA insertion into the genome of carcinoma cells constitute a highly specific genetic marker of tumor DNA. Recent data have shown that mutant DNA could be detected in the blood of patients with tumors and that the amount of circulating tumor DNA (ctDNA) was related to tumor dynamics [7]. This opens large perspectives for the use of ctDNA as a tumor biomarker in cancer patients [8]. In advanced stage cervical cancer, several studies have reported evidence of circulating HPV DNA (c-HPV DNA) [9], [10], [11] or circulating HPV RNA [12]. These studies showed that it was possible to detect viral nucleic acids in the blood of patients with cervical cancer, but due to lack of sensitivity [10], [13], [14] and questionable specificity [11], [13], the data was of little clinical impact. In this context, we designed a study to assess the value of insertional mutation of HPV DNA as a marker for the detection and the quantification of ctDNA at diagnosis in the serum of patients with invasive cervical cancer. We also looked for variations of ctDNA concentration during the course of the disease. In addition, to compare the sensitivity of our original and specific assay to that based on the detection of HPV DNA sequences, we also looked for c-HPV DNA using primers located in the E7 HPV16/18 gene.

## Materials and Methods

### Patients and tumors

From a series of HPV16 (14 cases) or HPV18 (2 cases) cervical carcinoma accumulated between 2001 and 2011, we determined the viral insertion locus using the DIPS-PCR method [15]. In the 16 cases, serum specimens taken at diagnosis before treatment were available. In two of these cases, additional sequential serum specimens taken during the course of the disease were also available. Fourteen tumors were invasive squamous carcinoma and two were glandular. In accordance with French regulation, all patients were informed and did not object to their biological specimens being used for research. The viral load, in tumor biopsy specimens was assessed by quantitative PCR (qPCR) using primers designed in the E7 HPV16/18 gene, as previously described [6]. Since integrated HPV DNA sequences may present amplification at the insertion locus, mutational insertion copy number was also assessed by qPCR using primers and reagents provided in Table S1. *KLK3* gene status was taken as a two copies reference.

### DNA serum analyses

DNA was isolated from 200 µl of serum with QIAamp Min Elute Virus Spin Kit (Qiagen®, Courtaboeuf, France) following the manufacturer's instructions. The elution step was performed with 25 µl of supplied buffer. Isolated DNA concentrations were measured using a Real-Time quantitative PCR (RT-qPCR) system based on Long Interspaced Nuclear Elements (LINE) sequence detection [16]. Dilutions of normal human DNA were used as standards and PCR was performed in 25 µl with Sybr® Green PCR Master Mix (Applied Biosystems, Villebon-sur-Yvette, France), primers mix (1 µM) and 2 µl of eluted DNA with the following cycling conditions: 15 min at 95°C and 45 cycles (15 s, 95°C; 15 s, 61°C; 1 min, 72°C) followed by a dissociation stage (15 s, 95°C; 15 s, 60°C; 15 s, 95°C). RT-qPCR assay using Sybr®Green (Applied Biosystems) was designed to specifically amplify cell-virus junction DNA sequences (eluted DNA 2 µl) or HPV16 E7 DNA (eluted DNA 2 µl) with 400 nM of each primer in a final volume of 25 µl. Each set of primers was tested on serial dilutions (from 50 ng/µl to 0.5 pg/µl) of matching tumor DNA in Tris-EDTA Buffer with thermocycler conditions of 15 min at 95°C and 45 cycles (15 s, 95°C; 1 min, 62°C) followed by a dissociation stage (15 s, 95°C; 1 min, 60°C; 15 s, 95°C). Primer sequences and MgCl_2_ concentrations are given in the Table S1. The sensitivity of the PCR assay had to be equal or greater than 5 pg/µl to validate the primer sets. In order to increase the sensitivity of the detection, 10 replicates of RT-qPCR assay were performed on each serum specimen for the detection of ctDNA and of c-HPV DNA. The amount of ctDNA in 200 µl of serum corresponded to the sum of the ctDNA detected in each positive replicate. The concentration of ctDNA was finally expressed in copies/ml serum.

## Results

Clinically, FIGO stages were from Ib to IVa and one case was a pelvic relapse of cervical SCC (**Table 1**). Tumor size ranged from 10 to 120 mm. The available squamous cell carcinoma antigen (SCC) values ranged from 1.1 ng/ml to 17.4 ng/ml (threshold of positivity: 1.5 ng/ml). HPV DNA was integrated at different loci (**Table 1**). The viral load in tumor specimens varied from 0.5 to 160 HPV16 genomes per cell (0.8 10^6^ to 24 10^6^ HPV16 copies/µg DNA). The concentration of circulating host DNA ranged from 6 10^1^ to 77 10^3^ copies/ml serum (**Table 1**). We were able to detect ctDNA in 11 out of 16 patients diagnosed with invasive cervical cancer. Three negative cases corresponded to the early stage (Ib) carcinoma with a size ranging from 10 to 20 mm (**Table 1**). The serum concentration of ctDNA ranged from 5 to 890 copies/ml serum, representing from 0.01% to 25% of circulating host DNA. c-HPV DNA was detected in 13/16 cases, the three early stage cases remaining negative. Values ranged from 5 to 8.5 10^3^ copies/ml serum (**Table 1**). The three highest values (8.5, 3.5 and 3.4 10^3^ copies/ml) corresponded to cases with high viral load in tumor biopsy specimens, suggesting that DNA derived from episomal molecules was also detected by the assay.

**Table 1:** Circulating tumor DNA in cervical cancer patients.

Cases	Tumor stage (FIGO)	Tumor size (mm)	HPV type	SCC marker (ng/ml)	HPV integration sites	HPV-E7 load in tumor (copies/cell)	HPV insertion load in tumor (copies/cell)	Circulating DNA (copies/ml serum)		
								Host DNA	HPV DNA	Tumor DNA
N°1	Ib	10	16	NA	Xq21.31	17	1	19 10^3^	0	0
N°2	Ib	15	16	1.1	3q26.32	1	1	21 10^3^	0	0
N°3	Ib	20	18	NA	17q23.1	0.6	0.6	41 10^3^	0	0
N°4	IIa	35	16	NA	14q32.2	55	25	17 10^3^	770	790
N°5	IIb	42	16	1.9	15q23	5	1	40 10^3^	5	5
N°6	IIb	47	16	NA	3q21.3	88	12	7 10^3^	390	70
N°7	IIb	53	16	6.4	5p31	27	0.7	7 10^3^	5	5
N°8	IIb	54	16	15.4	17q25.2	160	79	5 10^3^	3.4 10^3^	890
N°9	IIb	55	16	2.8	18q21.33	2	2	6 10^1^	30	15
N°10	IIb	56	16	12.9	4q13	58	1	55 10^3^	3.5 10^3^	0
N°11	IIIb	55	16	11.6	1p22.1	2	3	21 10^3^	45	20
N°12	IIIb	74	16	17.4	2q22.1	1	1	32 10^3^	10	20
N°13	IVa	46	16	NA	17q21.31	94	51	66 10^3^	8.5 10^3^	810
N°14	IVa	62	16	7.3	Xq22.3	0.5	0.5	77 10^3^	15	0
N°15	IVa	120	18	NA	1p31.1	5	11	3 10^3^	500	680
N°16	relapse	27	16	3.9	Repeated sequences*	22	18	5 10^3^	30	25

Abbreviations: FIGO: International Federation of Gynecology and Obstetrics; SCC: squamous cell carcinoma associated antigen; NA: not available;

* Repeated sequences: Homology with the centromeric sequences of various chromosomes.

**Figure 1:**
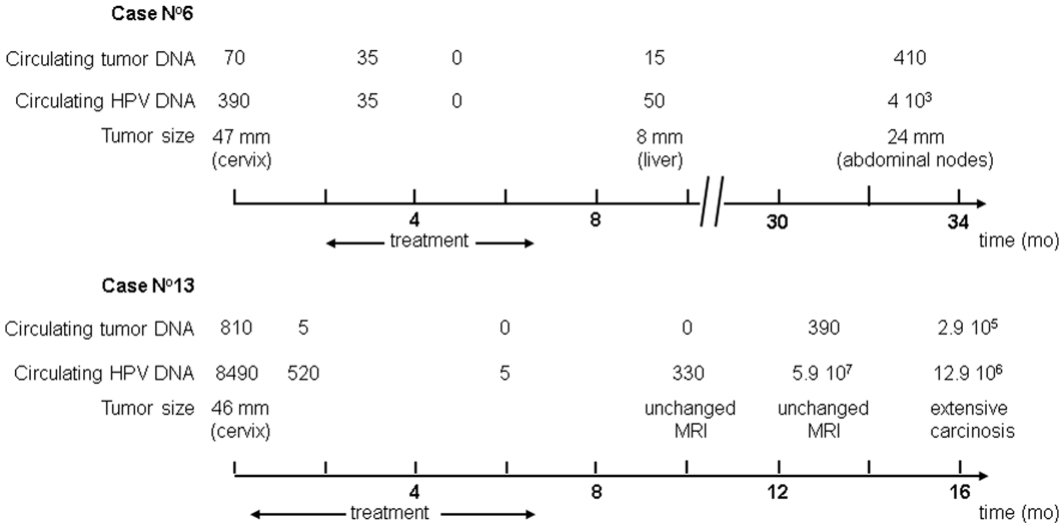
Circulating tumor DNA related to tumor dynamics in cervical cancer patients.

ctDNA dynamics were analyzed on sequential serum specimens in two patients. The first patient received combined chemoradiotherapy followed by utero-vaginal brachytherapy and surgery (**Figure 1**, case n°6). The ctDNA value decreased during treatment and was null by the end of therapy at which time a complete histological response was observed on the hysterectomy specimen. Two months later, the patient developed a 8 mm liver metastasis and ctDNA became detectable again. Increasing levels of ctDNA were later found when the patient developed abdominal node recurrence. The same dynamics were observed in the second patient treated exclusively with chemoradiotherapy (**Figure 1**, case n°13). Of interest during the follow-up, while the pelvic MRI showed no significant modification, a rise of ctDNA was observed. Soon afterwards the patient presented an abdominal relapse associated with high levels of ctDNA. The same dynamics were observed with c-HPV DNA.

## Discussion

We report here that, using HPV integration mutation as a molecular marker, ctDNA could be detected in the serum of most patients diagnosed with invasive cervical carcinoma over stage Ib and that the amount of ctDNA reflected tumor dynamics. Since the viral integration site is unique for each patient, this marker is highly specific, much more so than the SSC marker [17]. Regarding sensitivity, we could detect ctDNA in 11 out 13 (85%) patients with stage II–IV tumors, a rate largely higher than the 12% [9], 18% [13], 20% [14] and 50% [11] of positivity reported so far for c-HPV DNA. The practice of 10 replicates for the detection of ctDNA in each serum specimen may account for the increased sensitivity of our assay. For instance, in most of our positive cases, less than 50% of the replicates provided evidence of ctDNA. However, in 2 of the 13 cases with a tumour size >20 mm, no ctDNA was found whereas c-HPV DNA was detected. Both cases corresponded to tumors with low HPV insertion load (≤1 copy/cell). In contrast, cervical carcinoma cells frequently harbour free HPV genomes which are also released in the general circulation, thus increasing the rate of detection of circulating viral DNA. Nevertheless this asset may be balanced by a risk of false positivity. For instance, assays based on the detection on HPV DNA provided false positive rates of 13.5% in normal individuals and 33% in CIN3 patients [11]. In addition, frequent discrepancies between HPV genotypes found in cervical cancer and in the serum of the same patients were also reported [13]. These difficulties should be overcome by adequate controls of specificity and, for clinical purpose, c-HPV DNA analysis may represent a useful alternative where the identification or the use of viral-cell junction sequences is difficult.

Analysis of sequential serum specimens showed the dynamics of the results: the concentration of ctDNA decreased under treatment and increased at the time of relapse. The positivity of ctDNA preceded radiological relapse in one case and was associated with an 8 mm liver metastasis in the other one. Tumor DNA might be more easily released from tumor tissue corresponding to relapse or metastasis than to primary lesion. It might also derive from circulating tumor cells. A specific and quantitative molecular marker of ctDNA may be used to follow the course of cervical cancer. During initial treatment, ctDNA may help to assess disease prognosis and tumor sensitivity to therapy. Larger prospective studies will be necessary to validate this utility. During follow-up, ctDNA concentration could be a highly specific surrogate marker of minimal residual disease and subclinical relapse. This is of major importance in the perspective of specific anti-HPV therapy [18] whose potential efficiency is largely dependent on the tumor mass. These approaches could be easily extended to other types of HPV-associated tumors, anal canal and head and neck carcinoma, for example. Localization of the HPV integration site is not currently assessed in clinical practice. New technologies such as Next Generation Sequencing [19], however, should facilitate this determination and provide optimal molecular characterization of tumors to personalize the handling of patients. In addition, the sensitivity of the detection of ctDNA can be easily increased, merely by analyzing a larger amount of serum and/or by increasing the efficiency of the PCR assay, for instance using digital PCR [20].These advances will facilitate the assessment of the use of ctDNA in clinical oncology and will improve the biological follow-up of patients treated with cervical cancer.

## Supporting Information

Table S1 **Primers sequences and reagents for detection of ct-DNA.**
(DOC)
