# Supplementary material for: Human Papillomavirus Mutational Insertion: Specific Marker of Circulating Tumor DNA in Cervical Cancer Patients
Source: PLoS One. 2012 Aug 24;7(8):e43393. doi: 10.1371/journal.pone.0043393 (PMC3427328; doi:10.1371/journal.pone.0043393)
Supplement: Table S1 — Primers sequences and reagents for detection of ct-DNA. (DOC) [file pone.0043393.s001.doc]

| Cases | Primer sequences | Reagents |
| --- | --- | --- |
| N°1 | Forward AGACAATAGAAACTCAAGG | Sybr® Green PCR Core |
|  | Reverse CTGCATTTGGACTTACAC | Reagents Kit, 2.5 mM MgCl_2_ |
| N°2 | Forward CCAATGCCATGTAGACGA | Sybr® Green PCR Core |
|  | Reverse CCTAATCAGAAAACGGCAAC | Reagents Kit, 4 mM MgCl_2_ |
| N°3 | Forward GCCATTGCCTTTTATTATAGTTTCCA | Sybr® Green PCR Master |
|  | Reverse GAAAATGACAGTGATACAGGTGAAGA | Mix |
| N°4 | Forward GCTTCAGGTCATTCTCCAAAG | Sybr® Green PCR Master |
|  | Reverse GCAAAGGCAGCAATGTTAGC | Mix |
| N°5 | Forward TTTAACACAGGCAGAAACAGAGACA | Sybr® Green PCR Master |
|  | Reverse GGGTGGTGGCAGGAGTCA | Mix |
| N°6 | Forward TGAATATTTGGGCATCAGAGG | Sybr® Green PCR Core |
|  | Reverse CTTGGAGGTGCAGAAAGGT | Reagents Kit, 2.5 mM MgCl_2_ |
| N°7 | Forward ACCCTGTATTGTAATCCTG | Sybr® Green PCR Master |
|  | Reverse GTTTGTTAGGGGGATCTTGTAGT | Mix |
| N°8 | Forward CCAACATAGCGAAACCCCA | Sybr® Green PCR Core |
|  | Reverse TGCTTACAACCTTAGATAC | Reagents Kit, 4 mM MgCl_2_ |
| N°9 | Forward GATACATTGATTGATAAGGCATCG | Sybr® Green PCR Master |
|  | Reverse GACGACTATCCAGCGACCAA | Mix |
| N°10 | Forward CCACTACCTGTCTGTCTCC | Sybr® Green PCR Core |
|  | Reverse CTGTGGTAGAGGGTCAAGT | Reagents Kit, 2.5 mM MgCl_2_ |
